# Detecting Abnormal Vehicular Dynamics at Intersections Based on an Unsupervised Learning Approach and a Stochastic Model

**DOI:** 10.3390/s100807576

**Published:** 2010-08-11

**Authors:** Hugo Jiménez-Hernández, Jose-Joel González-Barbosa, Teresa Garcia-Ramírez

**Affiliations:** 1 Centro de Ingeniería y Desarrollo Industrial, Av. Pie de la Cuesta No. 702, Desarrollo San Pablo, Querétaro, Mexico; 2 Centro de Investigación en Ciencia Aplicada y Tecnología Avanzada. Cerro Blanco No. 141. Col. Colinas del Cimatario, Querétaro, Mexico; E-Mail: gonzbarjj@gmail.com; 3 Universidad Autónoma de Querétaro. Cerro de las Campanas s/n. Cerro de las Campanas, Querétaro, Mexico; E-Mail: garramt@hotmail.com

**Keywords:** abnormal activities detection, unsupervised learning, long binary strings

## Abstract

This investigation demonstrates an unsupervised approach for modeling traffic flow and detecting abnormal vehicle behaviors at intersections. In the first stage, the approach reveals and records the different states of the system. These states are the result of coding and grouping the historical motion of vehicles as long binary strings. In the second stage, using sequences of the recorded states, a stochastic graph model based on a Markovian approach is built. A behavior is labeled abnormal when current motion pattern cannot be recognized as any state of the system or a particular sequence of states cannot be parsed with the stochastic model. The approach is tested with several sequences of images acquired from a vehicular intersection where the traffic flow and duration used in connection with the traffic lights are continuously changed throughout the day. Finally, the low complexity and the flexibility of the approach make it reliable for use in real time systems.

## 1. Introduction

Recently in most large cities vehicular thoroughfares have become extremely congested. These cities need more efficient monitoring systems which are capable of acquiring information, such as non-desirable driver behaviors, vehicle’s crashes, or saturated avenues. The data collected can then be used for analysis regarding how to make improvements, nevertheless, the amount of data produced thereof is impossible to analyze through human resources. Today, approaches such as vision systems are primarily used to record data for areas where there are many reoccurring traffic related events [1]. However, the detection and the labeling of significant events are affected negatively by the environmental conditions and the complexity of the dynamics motion.

Several projects have been developed to deal with the monitoring and surveillance of specific scenarios. One of the first approaches is the research of Buxton [2] that establishes the foundation of a camera surveillance system based in Bayesian Networks. Kanade *et al.* [3] also proposed the structure of a surveillance vision system. They emphasised the balance between the computational resources and the complexity of the approaches used for analyzing video streams. Collin *et al.* [1] expanded the research of Kanade *et al.* [3] to multi camera surveillance systems. Later Oliver *et al.* [4] proposed a surveillance system framework based on Hidden Markovian Networks (HMN). None of these were scenario-oriented and were intolerant of outdoor environmentally changing conditions, describing only motion features. Other researchers have tried to create new approaches to interpret and record motion dynamics. The most noteworthy are the investigations of Rao and Mubarak [5]. They used a set of motion features in order to classify and group events by using motion patterns that describe actual actions. Lou *et al.* [6] also used a classifier and a metric, grouped by motion trajectories of traffic activities. Similarly, Hu *et al.* [7] proposed an approach based on statistical information of visual motion patterns. However, these approaches were limited to well-known scenarios and further limited to visual constraints as stationary behaviors.

All of the aforementioned, are characterized mainly by the classifications of motion where these are represented as any numerical representations. Using a classification process it is possible to discern distinctive numerical patterns. However, these approaches assume that the numerical representation used will be sufficient to capture all patterns of interest. Consequently, other researchers have been working on the development of better approaches to classify data, based on results by Shannon [8]. He exposed the problem of the information coding as well as the problem of providing a measure of the information. Furthermore, Kolgomorov [9] and Chaiti [10] expanded these ideas adding a stochastic approach. Mackay [11] discussed the need for a good representation for coding a problem. Finally, Brand and Kettnaker [12] provided a framework to develop optimum machine learning for modeling trajectories and inferring activity. These authors correctly emphasized that a proper representation of the problem was necessary before it could be classified or worked with.

Based on the hypothesis of an adequate coding scheme of data, an effective classifier can be developed. This research project presents an unsupervised approach for building automatic models of traffic flow for detecting abnormal vehicular behavior at intersections. This approach is based on detecting the most likely movement states of vehicle motions, and coding the historical motion as long binary strings. In addition, the properties of the space {0,1}*^n^* with a high dimensionality provided several properties that make it possible to reliably classify these strings and estimate the possible set of system states. Once the system states have been detected, a temporal relation is modeled as a time-state graph based on Markovian approach. An experimental model has been created to test the approach which consists of a camera located at the top of a monitoring tower at vehicular intersection. The results show the reliability of this approach in outdoors scenarios, even while climatic conditions are changing.

## 2. Motion Coding

In current literature there are several approaches for segmenting and locating moving objects [13,14]. Moreover, there is a compromise between the accuracy motion detection and the computational resources required. Seminal investigations [8,11] concluded that the information coding process and the set of operators, define the capabilities of classification. Herein is proposed a different approach for coding the motion performed by the objects; *i.e.*, the motion information is encoded as long binaries patterns.

**Figure 1.**
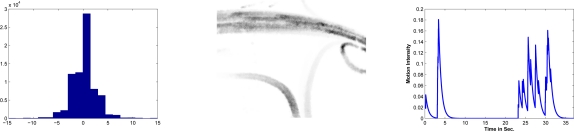
**(a)** Difference distribution of the first derivative from a pair of consecutive images; values are distributed mainly around the zero value. **(b)** Local historical motion for a short time instant. Gray zones represent zones with movement. Local historical motion captures the vehicle dynamic. **(c)** Decay motion function of one pixel where peaks show pixel occlusion and the vanishing peaks represent the historical memory of object that has been occluded within the pixel.

These patterns encode the temporal information of motion sources, which are the result of binarizing, the most recent historical motion in the scene. The process to generate these patterns consists of estimating the differences between the difference of each pair of consecutive images so that a derivative operator ∇ can be applied. Using the derivative image differences instead of simple image differences results in more complete data that considers the texture information and the local intensities dependences which in turn results in a more robust recording of small luminance variations. The approximation for estimating the image derivative depends on the texture levels of sequence analyzed. To approximate the image derivative, several approaches should be used. The most common of these are presented in Table 1. Consequently, given that an image sequence **I** = {*I*_1_, *I*_2_,...}, the intensity of changing regions can be detected by thresh-holding consecutive image differences as follows:

(1) $$M\left(I_{i},I_{j}\right)=\{\begin{array}{ll} 1 & \left|{\nabla I}_{j}-{\nabla I}_{i}\right|>\lambda_{d}\\ 0 & \text{Other case} \end{array}$$

where positions with one values represent areas with pixel changes greater than λ*_d_*. The value of λ*_d_* is calculated dynamically under the assumption of normality in the image difference distribution. Figure 1(a) illustrates the difference distribution of derivatives which becomes normal as can be noted when tested with Kolgomorov–Smirnov statistic [15]. Next, the probabilistic density function of images difference is modeled as a Gaussian *G*(∇*I_j_* − ∇*I_i_*;0, σ*_d_*) with the center at the origin: *i.e.*, values belonging to the Gaussian correspond to free motion zones, and consequently values distant to the origin, represent high probable motion zones. The value λ*_d_* is defined as *k* factor of σ*_d_*, which is in relation to the probability of belonging to zones free of movement. The Gaussian parameters are estimated with EM algorithm [16] under the assumption of incomplete data as follows:

$$\hat{\theta }=\left({\hat{\mu }}_{i},{\hat{\sigma }}_{i}^{2}\right)$$

where

$$\begin{array}{lll} {\hat{\mu }}_{i} & = & \rho_{1}{\hat{\mu }}_{t}+\left(1-\rho_{1}\right)x_{i}\\ {\hat{\sigma }}_{i}^{2} & = & \rho_{2}{\hat{\sigma }}_{t}+\left(1-\rho_{2}\right){\left({\hat{\sigma }}^{2}-x_{i}\right)}^{2}\\ & & \text{for}\;\rho_{1}\;\text{and}\;\rho_{2\;}\text{convergence constants}. \end{array}$$

The use of EM algorithm is to reduce the computational complexity by sampling the information from the images. This is especially useful when the image dimensions have high resolutions, or when available computer resources are limited. The values of ρ_1_ and ρ_2_ constants are defined in function of the number of samples used to estimate the parameters. Experimentally, we define ρ_1_ = ρ_2_ = 1 − (0.1*n*)^−1^; *i.e.*, the values are defined as the ten percentage of the total pixels *n* involved in the calculation of the parameters.

In fact, *M*(*I_i_*,*I_j_*) function represents the majority of changing zones. When objects are flat or have the same color, only the object borders are denoted. Then, using the cumulative short time instants, the historical of the differences encodes the displacement of objects. The presence of motion at images sequence is represented as a decay function of time as follows:

(2) $$M_{t}\left(I_{t}\right)={\rho M}_{t-1}\left(I_{t-1}\right)+\left(1-\rho \right)M\left(I_{t},I_{t-1}\right)$$

with ρ as a decay factor. The normalized values in *M_t_*(*I_k_*) are related with the probability of the presence of motion in the near past history. The binary motion pattern, in a particular instant *t*, is the result of selecting the most probable regions with motion. Each binary motion pattern is denoted as $M_{t}^{b}(I_{k})$ and is the result of thresholding Equation (2) using the concavity change position as the threshold which is denoted by λ*_m_*. Figure 1(c) illustrates the decay function of the most recent historical motion for one pixel. The sudden excitation time instants represents the recent objects that occlude the pixel. The threshold λ*_m_* and decay factor ρ determine the historical time-length of movement. Both have direct relation with frame rate acquisition.

Exploiting spatial information of historical motion, the array of pixels that conform each $M_{t}^{b}(I_{k})$ is considered to be an eight graph connected relation. Under this assumption, a morphological filter is applied to enhance and dismiss the noise effects. This filter emphasises in the high-connected zones; the opening morphological filter [18] is used to eliminate isolated regions and spurious motion zones. The properties of the structural element define the type and intensity of the noise that would be dismissed. However, one must be careful with the shape and geometrical properties of the structural element because it would deform the objects without considering the deformation caused by the camera perspective.

Finally, the motion pattern $M_{t}^{b}(I_{k})$ encodes the motion spatial relationships where each motion pattern is associated to a particular long binary string *s_t_*, with a *v*(*M*) transformation. This transformation maps from image dimension *p* × *q* to a binary string of dimension 1 × *n*, where the string dimension resulted would be *n* ≤ *p* × *q*. The dimensionality reduction is useful when someone wish to make a subsampling from $M_{t}^{b}$ image avoiding the use of extra computational resources. The existence of *v*(*M*) implies the inverse; *i.e.*, the function *v*^−1^(*M′*) Namely, map function can retrieve the spatial structure of coding from a binary string to image binary pattern.

## 3. Learning States Based on Viscous Morphological Reconstruction

In this section we present a novel approach to learn and estimate the states of the system. The information is encoded as elements of high-dimensionality binary space.

### 3.1. Binary Spaces with High Dimensionality

A binary space with a high dimensionality has several properties [19] that makes the development of classifiers reliable. One of the most important properties is related to the probability function density of the distance between each element under *L*_1_ metric [20]. Then, the difference of two strings in a binary space of *n* dimensions is considered as follows:

(3) $$-\left(s^{1},s^{2}\right)=\{\begin{array}{ll} 1 & \text{if}\;s_{i}^{1}=s_{i}^{2}\\ 0 & \text{Other case} \end{array}$$

Consequently, the norm of a binary string are defined as

(4) $$\left|s\right|=\sum_{i=1}^{n}s_{i}$$

Using last two definitions, the distance of two binary strings is defined as

(5) $$d\left(s^{1},s^{2}\right)=\left|s^{1}-s^{2}\right|$$

The distance distribution among all elements of the space has a binomial distribution $d(s^{i},s^{j})∼\left(\begin{array}{l} n\\ d \end{array}\right)=\frac{n!}{d!(d-1)!}$ under *L*_1_ metric [19], where *n* is the dimensionality space and *d* distance between two strings. When *n* dimension increases, the majority of binaries strings have a distance near to $\frac{1}{2}n$. The binaries strings located at a distance, among them, near to $\frac{1}{2}n$, are considered orthogonal. The orthogonality degree of a pair of binary strings is measured as the difference of the distance between them and $\frac{1}{2}n$, which is denoted by

(6) $$\phi_{n}\left(s^{i},s^{j}\right)=\left|\frac{1}{2}n-d\left(s^{i},s^{j}\right)\right|$$

which is zero when *s^i^* and *s^j^* are completely orthogonal and greater than zero when they become less orthogonal. Then, when *n* increases, the majority of elements of binary space become orthogonal; *i.e.*, any pair of elements in {0,1}*^n^* randomly selected has a high probability degree to be orthogonal. In addition, other useful operators are the intersection and the complement, which are defined for a given pair of binaries strings as follows:

(7) $$\begin{array}{r} \wedge :{\left\{0,1\right\}}^{n}\times {\left\{0,1\right\}}^{n}\to {\left\{0,1\right\}}^{n}\\ \text{where}\wedge \left(s^{i},s^{j}\right)\to s^{k}\;\text{such that}\;s_{p}^{k}=\{\begin{array}{ll} 1 & s_{p}^{i}=1\;\text{and}\;s_{p}^{j}=1\\ 0 & \text{Other case} \end{array} \end{array}$$

and the complement,

$$-:{\left\{0,1\right\}}^{n}\to {\left\{0,1\right\}}^{n}\;\text{where}\;\bar{s^{i}}=s^{j}\;\text{such that}\;s_{p}^{k}=\{\begin{array}{ll} 1 & s_{p}^{i}=0\\ 0 & \text{Other case} \end{array}$$

Both operators will be used at the following sections to establish a measure criterion among binary moving patterns.

### 3.2. Morphological Viscous Consistency

**Figure 2.**
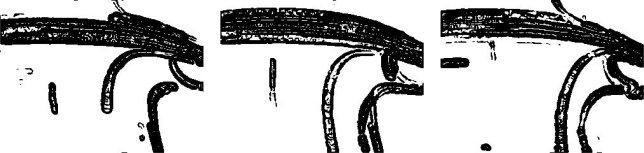
Samples of binary motion patterns; these patterns are continuously changing and their deformations are affected by the motion performed by the objects as can be noted in this sequence.

For each time instant a binary pattern $M_{t}^{b}(I_{t})$ is calculated. Then the approach consists of discovering different significant states among motion binaries patterns $M_{t}^{b}(I_{t})$. These states in turn lead to the main morphological properties of motion binaries patterns. However, these patterns are continuously changing as affected by the motion flow direction of the object involved at scene, see Figure 2. Motion flow provides information about the motion dynamics. Nevertheless, each binary motion pattern $M_{t}^{b}(I_{t})$ is affected by noise effects and environmental conditions; it causes the motion zones to be labeled incorrectly. This is considered to be a connectivity task which, using $M_{t}^{b}(I_{t})$, groups those regions that represent motion trends performed by the objects. Consequently, the binary historical motion of a particular object at specific time instant is represented as a connected region.

Given a pair of consecutive patterns, they are similar except that they include motion trend information. The highly connected areas in *t* and *t* + 1 represent motion dynamics of particular objects. The noise effects are a consequence of binarizing process, causing that motion trend to be a disconnected component. Real motion patterns are not well denoted because they may exist in small disconnected zones. These zones are hard to group as a part of binary historical motion pattern; also, they may present zones affected by noise. Then, the discarding of small noise-motion zones and addition of isolated connected motion zones become hard to perform. In [21] it is noted that there are several morphological approaches. However, many of them are context related and geometrically dependent. Further, many investigations [22–24] model the object motion at scene as optical flow, but they are computationally expensive. Combining both approaches, the motion object as a fluid connected surface can be considered. Under this assumption, the motion zones represent a viscous lattice [25]. The viscous lattice provides a framework where the connectivity is modeled as a fluid; what’s more, the historical motion represents motion zones as connected zones. This approach consists of managing each $M_{t}^{b}(I_{t})$ as a connected viscous lattice. Then, to group by the connected zones, a viscous filter is used where it analyzes each motion pattern, with the advantage that a viscous filter mixes up the closing and opening operators, instead of other approaches that consider each operator independently [25].

**Figure 3.**
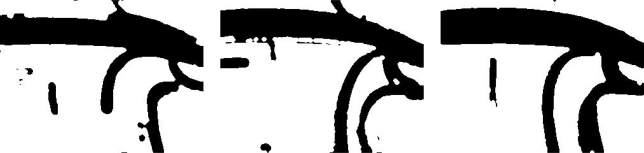
Samples of binary motion patterns after applying viscous filter; it was noticed that holes have been filled, noise effect has been dismissed and motion trend has been grouped in one motion blob.

For each binary pattern $M_{t}^{b}(I_{t})$, the associate lattice must be analyzed, because all connected areas represent motion zones or noise motion effects. To distinguish motion zones from noise zones, it is assumed that noise effects could be differentiated from lonely motion zones. The noise effects would be characterized by λ_1_, where λ_1_ is a morphological criterion. In the same way, motion zones do not necessarily become connected. Then, it is assumed similarly that λ_2_ is a morphological criterion of isolated motion zones. The λ_1_ and λ_2_ must be restricted to the constraint of λ_2_ ≥ λ_1_. Summing up these assumptions, a model as a connectivity problem is obtained. Later, considering $M_{t}^{b}(I_{t})$ as a viscous connected space, the opening and closing viscous operators can be defined as follows:

(8) $$\begin{array}{l} {\tilde{\gamma }}_{\lambda_{1},\lambda_{2}}\left(f\right)=\delta_{\lambda_{1}}R\left(\varepsilon_{\lambda_{1}},\varepsilon_{\lambda_{2}-\lambda_{1}}\varepsilon_{\lambda_{1}}\left(f\right)\right)\\ {\tilde{\phi }}_{\lambda_{1},\lambda_{2}}\left(f\right)={\tilde{\varepsilon }}_{\lambda_{1}}*R\left(\delta_{\lambda_{1}}\left(f\right),\delta_{\lambda_{2}-\lambda_{1}}\delta_{\lambda_{1}}\left(f\right)\right) \end{array}$$

where δ is the dilation operator, ε is the erosion operator, and *R*(*I*,*M*) is the reconstruction operator [18]. Lonely motion zones could be grouped and the noise motion effects are dismissed, therefore resulting in well-defined motion patterns. The viscous operators dismiss noise effects smaller than λ_1_ and group the motion zones that become isolated by almost λ_2_ connection criterion. The motion patterns, after filtering, become connected, ascertaining the principal motion flow and ignoring the noise effects, as noted in Figure 3. Consequently, a particular pattern is represented as $M_{t}^{∼}$ for a particular time instant *t*.

### 3.3. Similarity Measure and Learning Scheme

**Figure 4.**
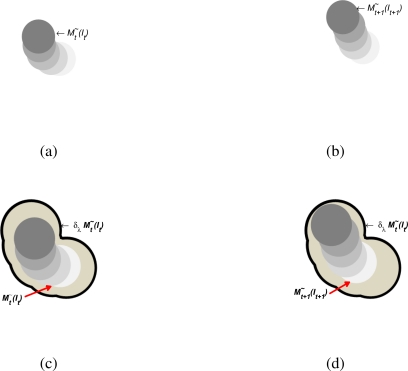
Similarity measurement scheme; **(a)** and **(b)** are two consecutive binary motion patterns which are presented at time instant *t* and *t* + 1; in **(c)** the dilation of *t* is illustrated; after **(d)** the dilation of motion pattern $M_{t}^{∼}(I_{t})$ is superposed on the $M_{t+1}^{∼}(I_{t+1})$ pattern.

Given a couple of motion patterns $M_{t}^{b}(I_{t})$ and $M_{t+1}^{b}(I_{t+1})$, the connected zones in $M_{t+1}^{b}(I_{t+1})$ can be discovered at the intersection of viscous opening applied to both patterns. The viscous motion patterns are denoted by $M_{t}^{∼}(I_{t})$ and $M_{t+1}^{∼}(I_{t+1})$. The viscous version group of each motion zone becomes greater than λ_1_ and isolated by a λ_2_ + λ_1_ criterion. This permits one to relate motion zones that are consistent with the source of motion and are not connected with representative motion patterns. This is especially important; in fact, the intersection of current viscous motion binary patterns and a dilated viscous motion binary pattern would be fully covered with all motion zones of the motion sources and isolated with zones located in different position zones, as in Figure 4. The covering of $M_{t+1}^{∼}(I_{t+1})$ via the dilation of $M_{t}^{∼}(I_{t})$ provided a criterion to decide when two different patterns belong to the same historical motion trend, where the dilation uses a λ*_m_* criterion that depends on the motion trends of the scene. The concept of similarity is viewed as the possibility to transform via dilate operator in a given viscous pattern to other viscous patterns, as follows:

Let a pair of binary patterns $M_{i}^{∼}$ and $M_{j}^{∼}$, verify this if $M_{j}^{∼}$ pattern is similar to $M_{i}^{∼}$ pattern. The pattern was dilated and measured the similarity to the $M_{j}^{∼}$ pattern. The similarity, in this sense, is achieved as summation of the overlapped motion zones and non-overlapped motion zones between reference binary pattern $M_{i}^{∼}$ and test binary pattern $M_{j}^{∼}$, as follows:

(9) $$d\left(M_{i}^{∼},M_{j}^{∼}\right)=a_{1}(\text{max}\{\left|M_{i}^{∼}\right|,\left|M_{j}^{∼}\right|\}-\left|M_{i}^{∼}\cap M_{j}^{∼}\right|)+a_{2}\left|M_{i}^{∼}\cap \bar{M_{j}^{∼}}\right|$$

The last expression becomes a formal metric [20] as shown in Appendix. Then, this expression quantifies the degree of similarity between a base binary pattern and a tested binary pattern. The first term measures the degree of belonging to the base pattern. When both patterns are exactly the same, the difference becomes zero; in other words, the value becomes greater than zero if there are some differences. The second term quantifies the degree of zones that do not fit with the base pattern. This is useful to discover when two patterns would be distinct even if they have a high intersection degree. Constants *a*_1_ and *a*_2_ are related with the weight of each term; this would be useful in situations where there is a need to penalize or permit some particular patterns, but for our purposes *a*_1_ = *a*_2_ = 1.

In addition, other useful measure named as dissimilarity were derived which conforms with the second last term of similarity measure. This measure quantifies the dissimilarity degree. When this measure becomes greater, the two patterns are too dissimilar because the negative version has a high intersection degree. When it becomes zero, the patterns are not dissimilar. Formally, it is defined as follows:

(10) $$\vartheta =\left(M_{i}^{∼},M_{j}^{∼}\right)=\left|M_{i}^{∼}\cap \bar{M_{j}^{∼}}\right|$$

that represents a formal metric as is seen in Appendix. Based on the last two similarity criteria, it is defined as an one forward-pass learning scheme to discover and identify the different motion patterns. The motion pattern classes are built with a set of motion patterns that could be considered as part of a base pattern. Then, given a set of motion pattern classes Σ = {*p*_1_, *p*_2_, . . ., *p_m_*}, the criterion is defined as the minimum distance of Equation (9) from the tested pattern to each class *p* and the distance must have almost λ*_th_* similarity degree; *i.e.*, binary motion pattern $M_{j}^{∼}$ belongs to *p_i_* if this is verified it will be:

(11) $$d\left(p_{i},M_{j}^{∼}\right)=\text{min}\left\{d\left(p_{i},M_{j}^{∼}\right)\right\}\;\text{and}\;d\left(p_{i},M_{j}^{∼}\right)\leq \lambda_{\mathrm{th}}$$

The value of λ*_th_* is determined by the dimensionality of binary space where motion patterns are coded. The threshold is based on the distance distribution and the orthogonality property as follows: $\lambda_{\mathrm{th}}=\frac{1}{2}\mathrm{nk}$; where *k* ∈ [0,1] and *n* is the space dimensionality of coding space. The value adopted by *k* defines the orthogonality degree of dissimilarity for a given pair of binary patterns.

### 3.4. Motion Patterns States

The process of relating two binary motion patterns plays a fundamental role in discovering the possible binary motion states on the scene. The approach uses an unsupervised one forward-pass learning approach for discovering the system states. Each different learned class represents a possible system state that encapsulate a set of similar binary motion patterns.

Then, for a given images sequence {*I*_1_, *I*_2_,…, *I_n_*} and its binarized motion patterns associated $\left\{M_{1}^{∼},M_{2}^{∼},\ldots ,M_{n}^{∼}\right\}$, the starting point consists in initialize a set of representative classes as an empty set Σ = {ε}. The learning process consists on discover the differents scene classes by grouping each binary motion pattern. The learning process is based on a variation of *k* neighborhood algorithm. The approach uses the first binary motion pattern as the base of the first class $p^{1}=M_{1}^{∼}$, updating Σ = Σ ∪{*p*^1^}. The similarity among successive binary motion patterns and the classes Σ is performed. Whenever there is no a similar class pattern and the dissimilarity measure ϑ is significant, a new class pattern *p^j^* is created and Σ is updated as Σ = Σ ∪{*p^j^*}. In another case, the most similar class *p^i^* is updated using current motion binary pattern $M_{j}^{∼}$. Class motion pattern *p^j^* is updated via Φ operator. The updating operator is usually defined as the binarization of probability for each component of the binary string to be a 0 or 1 value; *i.e.*, for a given pattern *p^j^* where each component $p_{k}^{i}=\text{max arg}\;P_{k}(\{0,1\})$.

The automatic discovery of the number of the states is not an easy task. In this case, it was assumed that the motion behavior is well-structured and distinguishable, each one to each other’s motion dynamics. Under this assumption, the different motion patterns at the scene would be captured with the last learning method. Nevertheless, as it is noteworthy in seminal work [26], there is no general criterion for classifying a set of data. Consequently, a good criterion may be proposed to estimate when the learning process has reached convergence.

**Figure 5.**
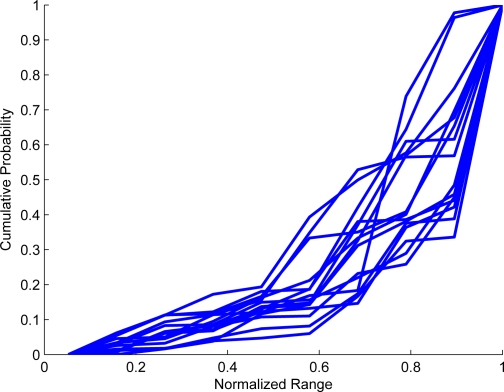
Several cumulative pdf of the number of learned states. At beginning, each pdf has an uniform distribution. Later, it is noticed that the pdf become quite similar as logarithmic distribution. Whenever a pdf distribution becomes as a cumulative log, it is possible to infer that a convergence is reached.

**Figure 6.**
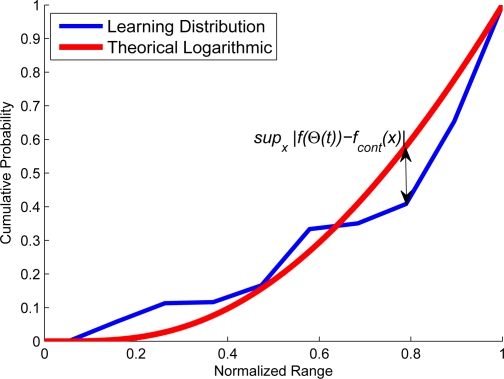
The number of states of the system reach the convergence when the pdf of the number of learned states becomes similar to an exponential pdf. The criterion used to test when learning pdf have become exponential is the Kolgomorov–Smirnov test, which is the supremum of the differences between the cumulative number of learned pdf and the cumulative exponential pdf.

**Figure 7.**
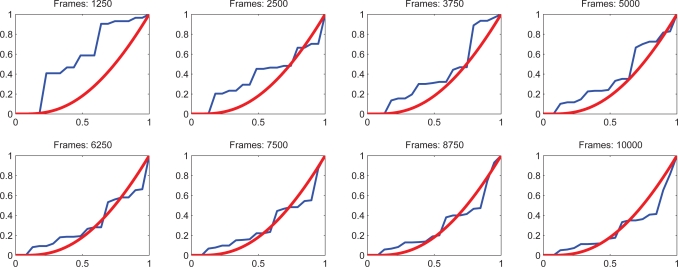
The theoretical cumulative pdf for an exponential is denoted by red line; in contrast, the learning cumulative pdf is denoted in blue. At the beginning the cumulative pdf behave as an uniform distribution. Afterwards, the number of states becomes constant with a exponential cumulative distribution.

Let Θ(*t*) = |Σ*_t_*| be a function that shows the number of classes at specific time *t*. The nature of function will determine the learning behavior. Experimentally, as it is noticed from Figures 4 and 5 that when there is a structured motion, the probability density function (pdf) of Θ(*t*) becomes a logarithmic function; in non-structured scenarios where the stability is not reached at Θ (*t*), the pdf tends to be a line. The proposal consists of matching the pdf of Θ(*t*) with an exponential distribution. The pdf of Θ(*t*) is denoted by *f* (Θ(*t*)). Consequently, when *f* (Θ(*t*)) has exponential distribution, it would be concluded that the learning process has reached stability, capturing the motion dynamics of scenario. The Kolgomorov–Smirnov test [15] is used to verify when *f* (Θ(*t*)) becomes exponential. The advantage of using this test is that it includes an uncertainty measure related to the probability that *f* (Θ(*t*)) becomes the desired pdf. This is

(12) $$\psi \;=\;\underset{x}{\text{sup}}\left|f\left(\Theta \left(t\right)\right)-f_{\mathrm{cont}}\left(x\right)\right|<{\mathrm{KS}}_{\mathrm{conf}}$$

where *f_cont_*(*x*) is a contrast function that need to be matched with *f* (Θ(*t*)), KS*_conf_* is a confidence index of the probability that *f* (Θ(*t*)) has the same distribution that the contrast function and the sup is the supremum operator. Figure 6 shows the Kolgomorov–Smirnov measure which represents the maximum distance between the cumulative pdf and the contrast of pdf. In addition, Figure 7 shows several runs. The time when *f* (Θ(*t*)) becomes exponential indicates that the learning process has reached stability, *i.e.*, it has captured the most significant motion dynamics. There would be some motion dynamics that were not learned, but it does not affect the performance. In essence, the unlearned dynamics do not correspond to the normal dynamics.

### 3.5. Abnormal Motion Detection

An abnormal motion behavior happens when there is no a model to explain the current information acquired; *i.e.*, abnormal motions are represented by dynamics with low probability. They are not modeled by the system. Then, formally, an abnormal behavior is denoted as follows:

(13) $$c\left(M_{i}^{∼},p_{j}\right)=\{\begin{array}{ll} 1 & \text{if}\;d\left(p_{i},M_{j}^{∼}\right)=\text{min}\left\{d\left(p_{i},M_{j}^{∼}\right)\right\}\wedge d\left(p_{i},M_{j}^{∼}\right)\leq \lambda_{\mathrm{th}}\wedge \vartheta \left(p_{i},M_{j}^{∼}\right)<\lambda_{\vartheta }\\ 0 & \text{Other case} \end{array}$$

where $d(p_{i},M_{j}^{∼})$ is the similarity measure, λ*_th_* is a similarity threshold of abnormal event detection, ϑ(*p_i_*,*M* *_j_*) is the dissimilarity measure and its threshold is λ_ϑ_.

## 4. Temporal Model

The abnormal motion pattern detection is focused on the recognition of the majority of common patterns; however, it does not check the time consistency such as abnormal dynamic detection process which identifies local historical motion that does not belong to the previous system states learned. Furthermore, it does not consider the time sequence dependencies among them. The Hidden Markovian Networks (HMN) [27,28] provided a framework to model sequence relationships as a probabilistic finite state machine. The relationships are interpreted as local temporal dependences. Therefore, a stochastic model based on HMN is built using the set of states Σ. These states conform to the set of observed symbols from the system. Afterwards, considering a sequence of symbols *S* = *s*_1_*s*_2_*s*_3_ … from Σ*, the function *s_i_*(*t*) denotes the symbol *s_i_* recognized at time *t*. Next, the probability of recognizing at two consecutive time instants *t* and *t* + 1 two arbitraries states *s_i_* and *s_j_* are denoted as follows:

(14) $$a_{\mathrm{ij}}=P\left(s_{j}\left(t+1\right)|s_{i}\left(t\right)\right)$$

Assuming that hidden states are the same as the visible states then *V* = Σ, which defines a identity bijection between each symbol in *V* and Σ such that:

(15) $$b_{\mathrm{jk}}=P\left(v_{k}\left(t\right)|v_{j}\left(t\right)\right)=1,\;\text{for}\;k=j$$

where *v*(*t*) is the observed symbol over time. Then, the probability of the system produce sequences of symbols *v* of length |Σ| is given by:

(16) $$P\left(v\right)=\sum_{r=1}^{\left|\Sigma \right|}P\left(v_{r}|w_{r}\right)P\left(w_{r}\right)$$

where *r* indexes a particular sequence. Considering an HMN of order 1, the last expression can be rewritten as:

(17) $$P\left(w_{r}\right)=\prod_{t=1}^{T}P\left(w(t\right)|w\left(t-1\right))$$

that is the product of *a_ij_* according to the sequence in question. Next, the probability densities for each pair of symbols is approximated with a Baum–Welch algorithm [29], which is an instance of EM algorithm [16]. Once the pdf is estimated for each pair of symbols *s_j_*(*t* + 1) and *s_i_*(*t*), the relation with *P*(*s_j_*(*t* + 1)|*s_i_*(*t*)) ≈ 0 corresponds to uncommon transitions and represents abnormal symbol relationship sequences. Finally, a stochastic automaton will be used to verify the temporal consistency of symbols sequences.

Then, a symbol sequence *S* = *s*_1_*s*_2_*s*_3_,…,*s_n_* is temporally valid if for each pair of consecutive symbols *s_i_* and *s_j_* if *p*(*s_j_*|*s_i_*) > λ*_p_*, where λ*_p_* is a probability threshold of a given pair of symbols which are temporally related. Furthermore, increasing the HMN order to *k* verifies the sequences of *k* length instead of using computational resources in the training step. The Markovian model provided a robust approach to parse sequences of symbols with the advantage that it verifies the most probable symbol sequences generated from the moving objects at the scene.

## 5. Experimental Model and Results

In this section, the experimentation and validation of the proposal approach is presented. The experimental analysis is oriented to a vehicular intersection where an stochastic model from motion vehicular flow is developed.

### 5.1. Experimental Model

The vehicular intersection scenario becomes interesting to this testing approach because the motion flow is time-dependent; *i.e.*, valid motion flows for a particular time instant become invalid for another time instant. Additionally, the duration of light changes and luminance scenario conditions does not remain constant during the time of day.

**Figure 8.**
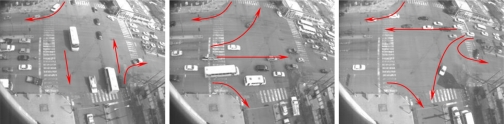
Different traffic flow states observed at the intersection. The motion flow pattern are time-dependent, and traffic lights remains changing over day time, becoming harder to model using only one camera information.

The experimental model for testing the proposed approach consists of a vision system mounted at the top of a tower. The tower is 25 meters tall and can cover the intersection completely. The camera has been configured to acquire 15 fps with a resolution of 320 × 240 pixels. The image processing is performed in a computer located at the base of the tower. Figure 8 shows the visual perspective of the camera view and the valid motion flows. The different traffic light variations are denoted by red arrows. The system is trained for modeling the vehicular dynamics at the intersection. The learning convergence at different time instants is illustrated in Figure 7. The system reaches the convergence at around 10,000 frames which is equivalent to approx 11 minutes. The convergence rate is defined at settings of *KS_conf_* value the significance of 0.9 to be a log pdf. In addition a similar time-period of 11 minutes was used to produce the significant symbol sequences to train the HMM. To summarize the parameters of the system these are illustrated in Table 2. The results herein are discussed in three stages. First are the comments regarding the accuracy of the resulting automaton, which is measured by comparing the events detected with a reference sequence as a ground truth. As a reference, an one hour time-length is used where the abnormal events are counted manually. Second, some of the learning processes are discussed and the automaton creation processes will be addressed. In the third stage, a measurement of the stability and robustness of the proposal will be discussed and the automaton is tested with a full journey of $5\frac{1}{2}$ hours. Finally, the results are discussed and commented upon.

### 5.2. Analysis of Results

In order to define a criterion by which to measure the efficiency of the proposal, the abnormal events that can be identified are grouped into three classes: (a) when there are historical motion patterns similar to the learned pattern, but with enough evidence for considering it as an abnormal historical pattern; (b) when there is unlearned state that represents the current historical motion pattern; and (c) when there is an invalid sequence of historical motion patterns over time.

Next, using the stochastic model generated by the approach, the measurement of the accuracy detection is performed. The results are compared with a reference ground truth. The ground truth consists of an one hour-length sequence where each abnormal event is detected manually. The sequence used as reference includes several luminance changes and different levels of traffic intensities. In addition, it must be considered that the automaton returns each frame that is not able to parse/recognize both the temporal model and the state model. Usually, an abnormal event is represented by several frames that are not recognized/parsed. Then, to associate it as an event in the ground truth and several abnormal frames detected by the systems, the frames are grouped in events with a temporal radius of ±2 s. The frames located temporally at a radius smaller that 2 s are joined as one event. When the temporal threshold covers the event it is manually detected. The detected event and the reference event are considered as the same.

**Figure 9.**
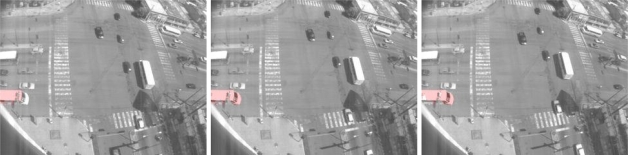
Events labeled as abnormal events. They represent motion that are not common and would represent events of interest.

**Figure 10.**
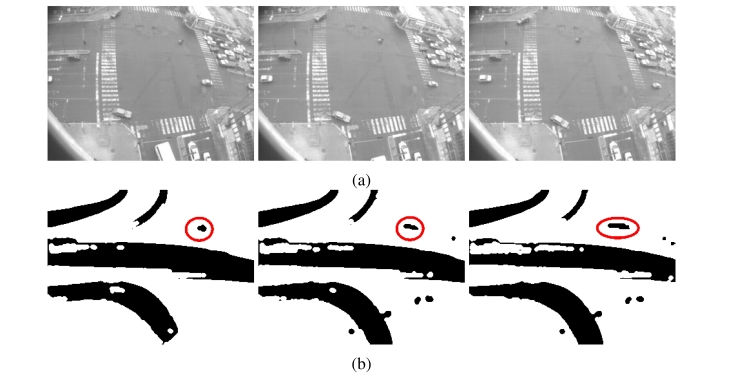
Abnormal event not recognized. The red circle show up the historical motion of the moving object. As it is appreciated, the object moving is small and the motion pattern does not have enough evidence to be considered as abnormal motion.

**Figure 11.**
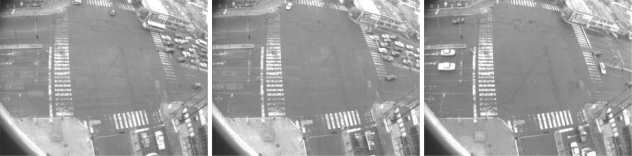
Frames not recognized as common states. They mainly belongs to scenes that do not have moving objects.

**Figure 12.**
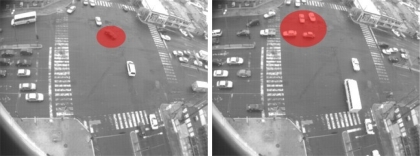
Frames sequences not be parsed. The objects motion perform uncommon dynamics, and in some cases results interesting to analyze.

**Figure 13.**
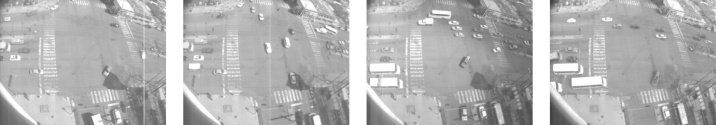
Sample of different environmental conditions at scene. The first two images represent situations where there are reflections caused by cars; the next two images represent rainy weather situations where the cloud sun occlusion causes changes in luminance conditions.

**Figure 14.**
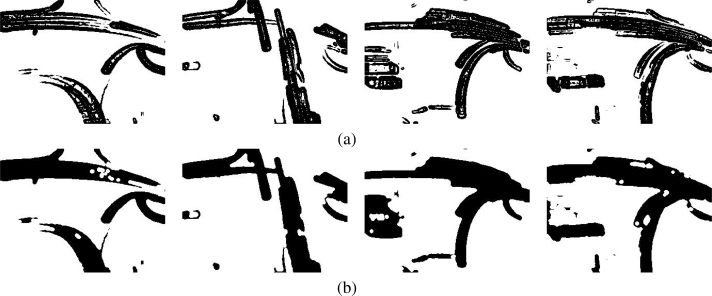
Historical motion of different time instants in different environmental conditions with/without connectivity analysis process: **(a)** Binary Historical Motion Patterns without connectivity analysis; motion patterns are not connected and present holes and noise motion; **(b)** Binary Motion History patterns with motion analysis or isolated areas have become connected and noise effect are dismissed.

Within these considerations, the accuracy is measured achieving an efficiency of 83.23% of detection, and a 16.77% of error. It is noteworthy that the level of accuracy is higher, even for the disturbances that present the scenario. The percentage of events that are not recognized as abnormal events are conformed by frames with small moving objects. The small objects become hard to characterize because the historical motion could be dismissed by the effect of the morphological filter, or the movement is not significant; an example is shown in Figure 9 where the motor cycle represents a small moving object. This can easily be considered as motion noise. In the figure it is noted that the motion historical pattern is not significant to infer if it corresponds to motion noise or to a motion object. However, a single measure of efficiency is not enough to characterize the reliability of the approach. There are more events detected than the number of the abnormal events in the ground truth. The results of the additionally detected events are shown in Table 3. These events correspond to the frames that are not recognized/parsed by the model. In an initial viewing these could be considered as false positives, but represent information of interest within the intersection; *i.e.*, in this particular case, the events labeled as abnormal correspond to motion patterns permitted but with low probability. As an example, Figure 10 represents a valid scene that presents motion in zones that expected to be free of movement. In the case of non-recognized states, the majority of these correspond to dynamics with low probability to be observed corresponding primarily to frames without significant moving objects, see Figure 11. In the case of unparsed events, they correspond to events that are not strictly abnormal, but they show information of uncommon situations that require attention, see Figure 12. Thus, the model results in not commonly observed efficiency in identifying motion patterns. In a second analysis, the approach is outdoor-oriented. To verify its robustness, the approach is tested with image sequences that presents luminance changes, caused by rain, sun occlusions or reflections, see Figure 13. The reflections are caused by car windows which would deform the motion objects; note the first two images on the left. The cloud occlusions and rain cause the luminance conditions to change quickly; note the last two images on the right in Figure 14, which are causing shadows or excessive brightness due to reflections. However, these disturbances do not negatively affect the historical motion patterns, see Figure 8 (a). The motion patterns are not affected by intensity variations of pixels and the motion historical trend captures the motion dynamic performed by the objects. The sudden luminance changes are supported because the historical motion is conformed by the probability of motion occurrence in a short period of time and inter-frame motion noise is discarded.

The binary motion patterns encode motion trend information; however, sometimes the motion trend is represented by isolated connected areas, or they could be affected by noise. To suppress these effects, the connectivity analysis based on a viscous lattice is applied, causing the noise effect and isolated zones to become grouped. The connectivity criteria in viscous filters are a pair of disks which are 3 and 5 pixel of radius. It was noted that the dimension and the structure are scenario and image resolution dependent. Figure 8(b) presents several frames that correspond to frames with several luminance disturbances after performing the connectivity analysis. The environmental disturbances have dismissed pointing out the object motion flow.

**Figure 15.**
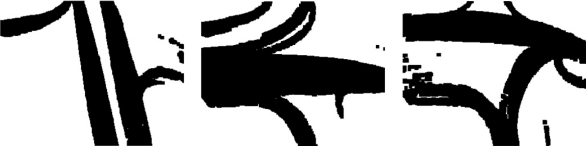
Some of different visual states discovered. States discovered that are highly correlated with common motion trends associate to each traffics light combination.

Once the learning process is applied until convergence, the system automatically identifies six different motion pattern flows. Some of these pattern are shown in the Figure 15 where it is noted that these patterns are highly correlated with the common vehicle motion flow, see Figure 8. The learning states are represented as binary patterns which are used as symbols to parse with an HMM. The learning process effectively exploits the dilation properties to group these by the binary motion patterns as a set of states. The learning process offers a robust approach to learn and automatically capture the vehicular dynamics.

**Figure 16.**
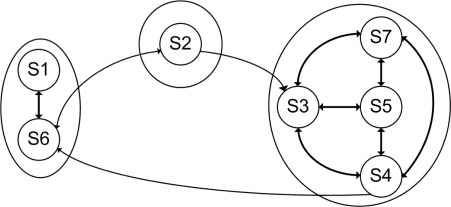
Graph model resulting from symbol sequences. The non-significant relations have been discarded. All states are reflexives.

**Figure 17.**
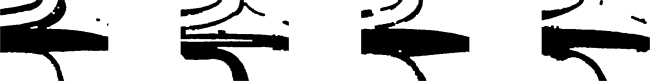
Different motion pattern estimated to represent the *G*_3_ state. Each pattern capture the most common historical motion trends performed by vehicles.

Next, using the following sequence of symbols of 11 minutes time-lengths, an stochastic model of scene using an one order HMN was built, see Figure 16. The graph model corresponds to the dynamic of the scenario. This model keeps the time state relationships. The non-significant relations between states are discarded and individual cyclic states relationships are not included for clarity. The graph has been grouped into three classes denoted by *G*_1_ = {*s*1,*s*2}, *G*_2_ = {*s*2} and *G*_3_ = {*s*3,*s*4,*s*5,*s*6}. Each class is associated with each one time-light configuration. As is noted, the relationship has been discovered following the common motion flow. There are traffic-light combinations that are represented by more than one state, as in *G*_3_ for instance. This obeys the assertion that there are several general patterns needed to model the traffic-light combination, see Figure 17.

**Figure 18.**
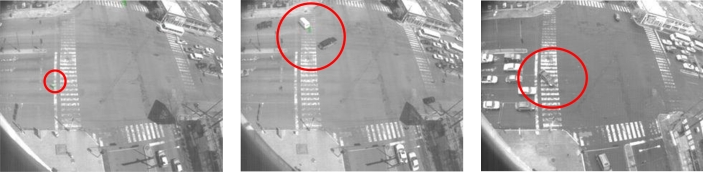
Samples of unrecognized historical motion patterns.

Finally, to test the robustness of the approach in outdoor scenarios, some of the results obtained from a journey are illustrated. Figure 18 presents some examples of frames that do not correspond to learned historical motion patterns. The first frame represents a pedestrian walking. In this case the small dynamics of a walking pedestrian was not learned so it is labeled as unidentifiable state. The second and third frames correspond to uncommon historical motion pattern too. In these cases, the dynamic corresponds to uncommon isolated historical motion observed. The uncommon motion patterns usually correspond to motions of interest. In this case both dynamics correspond to undesired motion behaviors that are adequately identified and logged.

**Figure 19.**
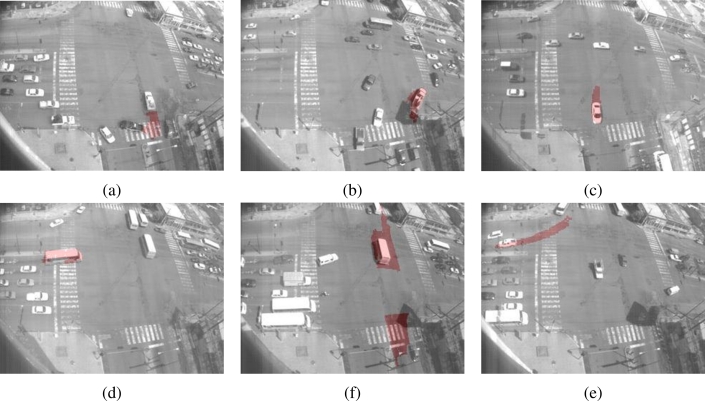
Abnormal vehicle behavior detection: forbidden turn **(a)** and **(b)**, open turn **(c)** traffic light forbidden **(d)** and lane invasion **(f)**.

The second type of abnormal events are shown in Figure 19. The first two frames, Figure 19(a,b) shows examples of an illegal turn. In Figure 19(a) the dark vehicle performed an illegal “U” turn. Next, in Figure 19(b) a truck performed a right turn. This turn is permitted temporally, see Figure 19(a), but in this frame it violates the traffic lights. Finally, Figure 19(c) shows an open turn. In this case, this vehicle must start turning before it actually did. Other examples of abnormal dynamic can be seen in Figure 19(d,e); both frames show a traffic light violation. In Figure 19(d), a bus is moving before red light. In Figure 19(e), a bus is moving even when the traffic lights are red. Finally, in Figure 19(f) the vehicle is executing a lane invasion where legally this turns must be executed only from internal lanes.

**Figure 20.**
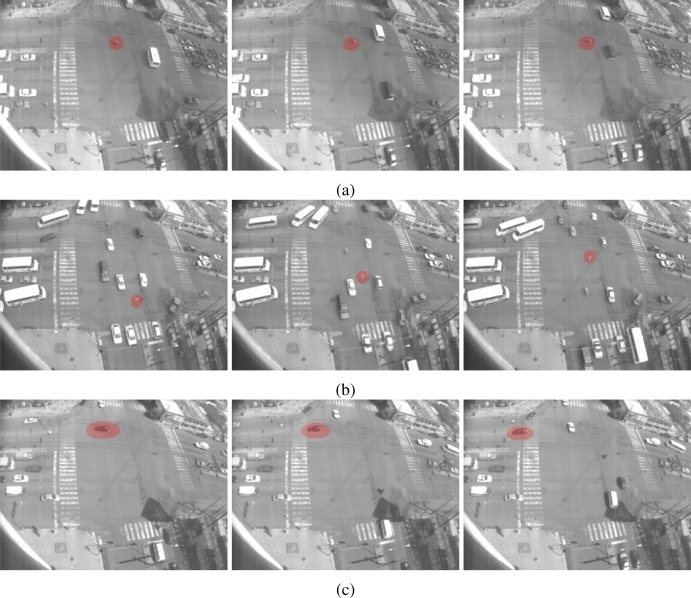
Samples of time inconsistencies detected with the HMM. In **(a)** a motorcycle goes into the main traffic flow; in **(b)** a pedestrian walking down in the middle of a traffic flow; and in **(c)** a vehicle passing by when the traffic light combination does not permit this circulation.

**Figure 21.**
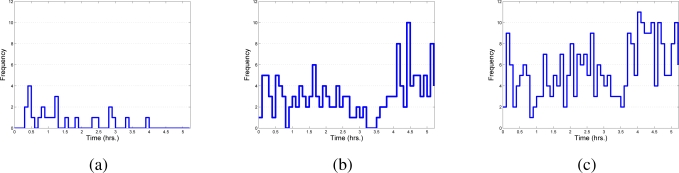
Distribution of detected events over time. In **(a)** the dynamic labeled is represented as abnormal, in **(b)** the events that represent unrecognized states, in **(c)** the events detected as time inconsistent are shown.

In the third group, certain images sequences violate the temporal dependencies. In Figure 20 some samples of abnormal sequences of images that have been detected are illustrated. In Figure 20(a) a motorcycle remains in motion even when the traffic light combination does not permit movement. In this sample, the motion state inferred is not consistent with the current motion flow observed but the sequences of states detected are not consistent, labeling them as abnormal. Figure 20(b,c) shows similar situations where a pedestrian is walking down the street in the middle of traffic flow and a vehicle is passing by yet there is inadequate traffic light indications to permit this. Finally, in Table 4 is a summary of the events detected by the system within the tested scenario. Figure 21 shows the distribution of the times of the different event recorded as aforementioned.

The approach herein and that of Brand and Kettnaker [12] were compared. First of all, their research deals with the problem of modeling activities in a scenario. They proposed an HMM model in order to explain the activities. They also tried to use a minimum number of expressions for the number of states that conform to the system. The minimum expression is denoted by the minimization of the entropy of each state. However, they do not deal with the problem of coding motion, assuming that the raw data encoded is provided depending on the scene, and they do not deal with the learning problem convergence. In the approach herein, these investigators focused on an adequate model for coding the information. This will be equivalent to the entropy minimization. But Brand and Kettnaker did not provide an scheme to encode the object’s motion, nor a criterion to reach the convergence for the learning of states. These two matters could cause the Brand and Kettnaker approach to present limitations in the learning stage. In addition, their research has been formulated to be off-line, unlike this approach which is developed to work with on-line systems. This conceptual difference makes the results of the present approach more efficient in discovering structured motion in the scenarios outlined.

The testing process performed showed some of the possibilities of the approach for detecting unusual events based on previous learning processes. The events detected correspond to the majority of real events; the rest of events detected correspond to motion patterns that are not abnormal, but are of interest. As noted, the proposed approach is capable of detecting events of interest. Moreover, semantic interpretation is left to the operator to make a final decision. Some other advantages include that the system is capable of inferring and learning a state system automatically, resulting a stochastic graph model of common motion observed. In this sense, based on the most likely motion pattern observed, the abnormal events are defined, as those motion vehicles which are not modeled by the proposal model. The vehicle dynamics focus of motion patterns that are not recognized could be of interest.

## 6. Conclusions

This investigation presents a novel automatic abnormal event detection approach based on a binary coding and a stochastic model. The proposal uses the advantages of coding the information in a long binary space and the connectivity analysis based on viscous lattices and morphological operators which conforms the learning process. These elements help to reveal a set of different states that model the motion dynamic in the scene. Afterwards, using the discovered states, an HMM approach defines a stochastic automaton of the time relationships of states. Motion coding was used to provide a different way to encode the information and discover the intrinsic dynamics using only the visual information. In addition, the motion process coding has been designed to tolerate several environmental disturbances such as shadows, rain, reflections or cloud occlusions. The criteria and measurements of similarity were defined, providing an adequate framework to develop pattern binary classifiers. The experimental results showed that in outdoors environments, the proposed system is capable of identifying uncommon motion events, even when the conditions are continuously changing. Furthermore, the low complexity of implementation makes it reliable to use this approach outdoors real time.

## Figures and Tables

**Table 1.** Different convolution approaches used to estimate the image derivative [17]. The use of one of them depends of scene conditions. Usually, derivative approaches used for border detection, results better descriptor of the texture of moving objects.

**Approach**	**Derivative Approximation**

Convolution Mask	Simple Derivative.
	Sobel Mask.
	Prewitt Mask.
	Laplacian Mask.
	Roberts Mask.
	Deriche Mask.

Morphological Operator	Inner Derivative.
	Outer Derivative.

**Table 2.** Table of parameters needed by the proposal.

**Parameter**	**Values**

**Motion Coding**	

∇	Sobel Mask
ρ_1_	1 *−* (0.1*n*)*^−^*^1^*
ρ_2_	1*−* (0.1*n*)*^−^*^1^*
ρ	1 *−* (5 * *f ps*)*^−^*^1^**
λ*_d_*	*k*σ for *k* = 3

**Learning States**	

λ_1_	disk of 3 size
λ_2_	disk of 5 size
λ*_th_*	${\frac{1\frac{1}{8}6400}{2}}^{***}=400$
*KS_conf_*	0.90
λ*_p_*	0.01

* where *n* is the number of pixels involved.

** where *f ps* is the number of frames per second acquired.

*** where 6400 is the number of pixels in images of 320 × 200 resolutions.

**Table 3.** Statistics of events recognized that are not considered as abnormal. These events provided information of low probable motion patterns.

**Description**	**Number of Events**
Events recognized as Abnormal	9
Events non recognized as an states	8
Events not parsed	29

**Table 4.** Statistics of event detected in the intersection.

**Description**	**Number**
Abnormal events detected	983 frames
Non-Recognized states	587 frames
Non-Recognized sequences of states	307 states transitions

## Appendix—Metric Proof

To prove that Equation (9) is a metric, this can be done in two stages. Each stage proves that each one term is a metric.

It is assumed that $d_{1}\left(M_{i}^{∼},M_{j}^{∼}\right)=\text{max}\left\{\left|M_{i}^{∼}\right|,\left|M_{j}^{∼}\right|\right\}-\left|M_{i}^{∼}\cap M_{j}^{∼}\right|$ is a metric. Then, by this assumption it can be verified that

1. $d_{1}(M_{i}^{∼},M_{i}^{∼})=0$; using a pair of binary patterns $M_{i}^{∼}$ and $M_{j}^{∼}$, we have that
  $$\begin{array}{ll} \text{iif} & d_{1}\left(M_{i}^{∼},M_{j}^{∼}\right)=0 \end{array}$$
  $$\begin{array}{lll} \text{By definition}\;d_{1} & & \\ & ↔ & \text{max}\left\{\left|M_{i}^{∼}\right|,\left|M_{j}^{∼}\right|\right\}-\left|M_{i}^{∼}\cap M_{j}^{∼}\right|=0\\ & ↔ & \text{max}\left\{\left|M_{i}^{∼}\right|,\left|M_{j}^{∼}\right|\right\}=\left|M_{i}^{∼}\cap M_{j}^{∼}\right| \end{array}$$
  (18) $$\begin{array}{ccc} \text{By definition of max} & & \\ & ↔ & \left|M_{i}^{∼}\right|=\left|M_{i}^{∼}\cap M_{j}^{∼}\right|\;\text{or} \end{array}$$
  (19) $$\left|M_{i}^{∼}\right|=\left|M_{i}^{∼}\cap M_{j}^{∼}\right|$$
  Next, making the substitution of the first one into the second one we have that $\left|M_{j}^{∼}\right|=\left|M_{i}^{∼}\right|$. Perhaps, under the identity of indiscernibles [30], we conclude that $d_{1}(M_{i}^{∼},M_{j}^{∼})=0$ iff $d_{1}(M_{i}^{∼},M_{i}^{∼})=0$.
2. $d_{1}(M_{i}^{∼},M_{j}^{∼})=d_{1}(M_{j}^{∼},M_{i}^{∼})$; using contradiction proof it is clear that
  (20) $$d_{1}\left(M_{i}^{∼},M_{j}^{∼}\right)\neq d_{1}\left(M_{j}^{∼},M_{i}^{∼}\right)$$
  is true, but, $\text{max}\left\{\left|M_{i}^{∼}\right|,\left|M_{j}^{∼}\right|\right\}=\text{max}\left\{\left|M_{j}^{∼}\right|,\left|M_{i}^{∼}\right|\right\}$ and $\left|M_{i}^{∼}\cap M_{j}^{∼}\right|=\left|M_{j}^{∼}\cap M_{i}^{∼}\right|$ then Equation (20) becomes false, consequently $d_{1}(M_{i}^{∼},M_{j}^{∼})=d_{1}(M_{j}^{∼},M_{i}^{∼})$ is true.
3. $d_{1}\left(M_{i}^{∼},M_{j}^{∼}\right)+d_{1}\left(M_{j}^{∼},M_{k}^{∼}\right)\geq d_{1}\left(M_{i}^{∼},M_{k}^{∼}\right)$; expanding each term and grouping show that
  $$\begin{array}{r} \text{max}\left\{\left|M_{i}^{∼}\right|,\left|M_{j}^{∼}\right|\right\}-\left|M_{i}^{∼}\cap M_{j}^{∼}\right|+\text{max}\left\{\left|M_{j}^{∼}\right|,\left|M_{k}^{∼}\right|\right\}-\left|M_{j}^{∼}\cap M_{k}^{∼}\right|\geq \\ \text{max}\left\{\left|M_{i}^{∼}\right|,\left|M_{k}^{∼}\right|\right\}-\left|M_{i}^{b}\cap M_{k}^{∼}\right|;\\ (\text{max}\left\{\left|M_{i}^{∼}\right|,\left|M_{j}^{∼}\right|\right\}+\text{max}\left\{\left|M_{j}^{∼}\right|,\left|M_{k}^{∼}\right|\right\})-\left(\left|M_{i}^{∼}\cap M_{j}^{∼}\right|+\left|M_{j}^{∼}\cap M_{k}^{∼}\right|\right)\geq \\ \text{max}\left\{\left|M_{i}^{∼}\right|,\left|M_{k}^{∼}\right|\right\}-\left|M_{i}^{∼}\cap M_{k}^{∼}\right|; \end{array}$$
  To simplify, it was assumed that the cardinality of each $\left|M_{i}^{∼}\right|$ be the same. Left side of the expression have three cases, when $\left|M_{i}^{∼}\right|$ patterns are completely non-overlapped, completely overlapped, or partial overlapped.
  - When they are completely non-overlapped, the left side can be rewritten as $(\text{max}\left\{\left|M_{i}^{∼}\right|,\left|M_{j}^{∼}\right|\right\}+\text{max}\left\{\left|M_{j}^{∼}\right|,\left|M_{k}^{∼}\right|\right\})-\left(\left|M_{i}^{∼}\cap M_{j}^{∼}\right|+\left|M_{j}^{∼}\cap M_{k}^{∼}\right|\right)\Rightarrow \left(k+k\right)-\left(0+0\right)\Rightarrow 2k$ and right side similarly is rewritten as $\text{max}\left\{\left|M_{i}^{b}\right|,\left|M_{k}^{b}\right|\right\}-\left|M_{i}^{b}\cap M_{k}^{∼}\right|\Rightarrow k+0\Rightarrow k$, which becomes true.
  - When they are completely non-overlapped, the left side can be rewritten as $(\text{max}\left\{\left|M_{i}^{∼}\right|,\left|M_{j}^{∼}\right|\right\}+\text{max}\left\{\left|M_{j}^{∼}\right|,\left|M_{k}^{∼}\right|\right\})-\left(\left|M_{i}^{∼}\cap M_{j}^{∼}\right|+\left|M_{j}^{∼}\cap M_{k}^{∼}\right|\right)\Rightarrow \left(k+k\right)-\left(k+k\right)\Rightarrow 0$ and right side similarly is rewritten as $\text{max}\left\{\left|M_{i}^{∼}\right|,\left|M_{k}^{∼}\right|\right\}-\left|M_{i}^{∼}\cap M_{k}^{∼}\right|\Rightarrow k+k\Rightarrow 0$, which becomes true.
  - When they are partially overlapped $\left(\text{max}\left\{\left|M_{i}^{∼}\right|,\left|M_{j}^{∼}\right|\right\}+\text{max}\left\{\left|M_{j}^{∼}\right|,\left|M_{k}^{∼}\right|\right\}\right)-\left(\left|M_{i}^{∼}\cap M_{j}^{∼}\right|+\left|M_{j}^{∼}\cap M_{k}^{∼}\right|\right)\Rightarrow \left(k+k\right)-\left(k_{1}\right)\Rightarrow 2k-k_{1}$ and right side similarly is rewritten as $\text{max}\left\{\left|M_{i}^{∼}\right|,\left|M_{k}^{∼}\right|\right\}-\left|M_{i}^{∼}\cap M_{k}^{∼}\right|\Rightarrow k-k_{1}$, which becomes true.

Then for (a), (b), and (c), the hypothesis becomes true.

Finally, for 1, 2 and 3 *d*_1_ is a formal metric.

Now, it was assumed that $\vartheta \left(M_{i}^{∼},M_{j}^{∼}\right)=\left|M_{i}^{∼}\cap \bar{M_{j}^{∼}}\right|$ is a metric. In the same way, it was assumed that we can probe the follows statements Then, by that assumption it can be verified that

1. $\vartheta \left(M_{i}^{∼},M_{i}^{∼}\right)=0$; for a given pair of binary patterns $M_{i}^{∼}$ and $M_{j}^{∼}$, we have that $\vartheta \left(M_{i}^{∼},M_{j}^{∼}\right)=\left|M_{i}^{∼}\cap \bar{M_{j}^{∼}}\right|=0$. Then the expression $\left|M_{i}^{∼}\cap \bar{M_{j}^{∼}}\right|=0$ is true only when $M_{i}^{∼}$ and $\bar{M_{j}^{∼}}$ become totally disjoint, that is, for a particular binary pattern $M_{i}^{∼}$, $\bar{M_{j}^{∼}}$ must be $\bar{M_{i}^{∼}}$; *i.e.*, $\vartheta \left(M_{i}^{∼},M_{j}^{∼}\right)=0\;\text{iff}\;\vartheta \left(M_{i}^{∼},M_{i}^{∼}\right)=0$.
2. $\vartheta \left(M_{i}^{∼},M_{j}^{∼}\right)=\vartheta \left(M_{j}^{∼},M_{i}^{∼}\right)$; which is true in sense $M_{i}^{∼}\cap \bar{M_{j}^{∼}}=\bar{M_{j}^{∼}}\cap M_{i}^{∼}$.
3. $\vartheta \left(M_{i}^{∼},M_{j}^{∼}\right)+\vartheta \left(M_{j}^{∼},M_{k}^{∼}\right)\geq \left(M_{i}^{∼},M_{k}^{∼}\right)$; expanding each term and grouping reveals:
  (21) $$\left|M_{i}^{∼}\cap \bar{M_{j}^{∼}}\right|+\left|M_{j}^{∼}\cap \bar{M_{k}^{∼}}\right|\geq \left|M_{i}^{∼}\cap \bar{M_{k}^{∼}}\right|$$
  which is true when they are completely non-overlapped because $\left|M_{i}^{∼}\cap \bar{M_{j}^{∼}}\right|=0$ and $\left|M_{i}^{∼}\cap \bar{M_{j}^{∼}}\right|=0$. As the same way, is true when are full overlapped because $\left|M_{i}^{∼}\cap \bar{M_{j}^{∼}}\right|+\left|M_{j}^{∼}\cap \bar{M_{k}^{∼}}\right|\geq \left|M_{i}^{∼}\cap \bar{M_{k}^{∼}}\right|\Rightarrow 2k\geq k$. When they are partially overlapped right side become maxima, when $M_{i}^{∼}=\bar{M_{k}^{∼}}$, at right side first almost be equal than $\left|M_{i}^{∼}\cap \bar{M_{k}^{∼}}\right|$ because $\left|M_{i}^{∼}\cap \bar{M_{j}^{∼}}\right|$ has the same cardinality.

Therefore by concluding, that by using 1, 2 and 3, $\vartheta \left(M_{i}^{∼},M_{j}^{∼}\right)$ is a formal metric.

Finally, any linear combination of two metrics as $d_{1}\left(M_{i}^{∼},M_{j}^{∼}\right)$ and $\vartheta \left(M_{i}^{∼},M_{j}^{∼}\right)$ is a metric too; which proves the hypothesis stated.
